# Use of Electroconvulsive Therapy for Catatonia in Down Syndrome Regression Disorder: A Systematic Review, Case Series, and Analysis

**DOI:** 10.1007/s10803-026-07500-3

**Published:** 2026-08-28

**Authors:** Seri Lim, Kaidi Kang, Elizabeth Oey, Stephanie Santoro, Catherine Franklin, James Luccarelli, Rafael Tamargo, Courtney Hamilton, Sarah Marler, Timothy Adegoke, Jo Ellen Wilson, Joshua Ryan Smith

**Affiliations:** 1 Division of Child and Adolescent Psychiatry, Department of Psychiatry and Behavioral Sciences, Vanderbilt University Medical Center at Village of Vanderbilt, 1500 21st Avenue South, Suite 2200, Nashville, TN 37212, USA; 2 Department of Biostatistics, Vanderbilt University Medical Center, 2525 West End Ave, Suite 1100, Nashville, TN 37205, USA; 3 Down Syndrome Program, Division of Medical Genetics and Metabolism, Department of Pediatrics, Massachusetts General Hospital, 55 Fruit Street, Boston, MA 02114, USA; 4 Queensland Centre of Excellence in Intellectual Disability and Autism Health, Mater Research Institute-UQ, The University of Queensland, Sir Fred Schonell Drive, Brisbane, QLD 4072, Australia; 5 Department of Psychiatry, Harvard Medical School, 24 Shattuck Street, Boston, MA 02115, USA; 6 Department of Psychiatry, Massachusetts General Hospital, 55 Fruit Street, Boston, MA 02114, USA; 7 Division of General Psychiatry, Department of Psychiatry and Behavioral Sciences, Vanderbilt University Medical Center at Village of Vanderbilt, 1500 21st Avenue South, Suite 2200, Nashville, TN 37212, USA; 8 Critical Illness, Brain Dysfunction and Survivorship Center, Center for Health Services Research, Nashville, TN 37212, USA; 9 Geriatric Research, Education, and Clinical Center Service, Veterans Affairs Tennessee Valley Healthcare System, Nashville, TN 37212, USA; 10 Division of Geriatric Psychiatry, Department of Psychiatry and Behavioral Sciences, Vanderbilt University Medical Center, 1601 23rd Ave South, Nashville, TN 37232, USA; 11 Vanderbilt Kennedy Center, Vanderbilt University, 110 Magnolia Circle, Nashville, TN 37203, USA

## Abstract

**Purpose:**

Down syndrome regression disorder (DSRD) is a condition occurring in young people with Down syndrome. It is associated with functional decline, new onset psychopathology, and increased support needs. DSRD is often associated with catatonia, for which electroconvulsive therapy (ECT) is an effective treatment. However, there are scant studies to guide best practice in this area.

**Methods:**

We report a case series of 5 patients who have received ECT at Vanderbilt University Medical Center. A systematic review and analysis were conducted, combining this case series with previously reported data in the literature.

**Results:**

This analysis included data from 27 patients, 5 from the VUMC case series and a further 22 from 14 studies. Combining all patients from case series and the systematic review, there were 17 pre-ECT and 15 post-ECT Bush Francis Catatonia Rating Scale (BFCRS) scores reported from 7 sites/studies. The mean pre-ECT BFCRS score from all patients was 17.40 (SD = 5.72) and the mean post-ECT BFCRS score was 4.40 (SD = 5.26). A generalized estimating equations analysis revealed that there was a significant difference in the expected BFCRS score before and after receiving ECT treatment (*β* = −13.00 with 95%, *p* < 0.001), suggesting that the ECT treatment had a significant effect on reducing the BFCRS scores. No severe adverse events were reported.

**Conclusion:**

ECT is associated with clinical improvement in patients with catatonia in the context of DSRD in this systematic review and analysis. More research is needed to provide a more robust evidence base.

## Introduction

Down syndrome (DS) is a neurodevelopmental disorder that occurs in 1 in 800–1000 live births with over 214,000 people with DS in the United States (De Graaf et al., 2015; Graaf et al., 2016). It is the most common cause of intellectual disability worldwide and is most frequently caused by trisomy of chromosome 21 due to nondisjunction or translocation events (Mircher et al., 2017). A possible complication of DS is Down syndrome regression disorder (DSRD), previously referred to as Down syndrome disintegrative disorder (DSDD). DSRD is a potentially life-threatening illness with newly developed consensus diagnostic criteria (Santoro et al. 2022a, b). DSRD is characterized by an acute or subacute loss of previously acquired skills, cognitive decline, affective instability, and language regression (Rosso et al., 2020; Santoro et al., 2023; Smith et al., 2024a). DSRD also includes symptoms of mood lability, new-onset insomnia, psychomotor slowing, mutism, and catatonia (Devenny & Matthews, 2011; Mircher et al., 2017; Worley et al., 2015). The etiology and mechanisms of DSRD are not well understood and have been hypothesized to include autoimmune dysregulation, psychosocial stressors, and co-morbid psychiatric disorders (Rosso et al., 2020; Santoro et al., 2024). Moreover, there is little literature which specifically addresses the treatment of DSRD; approaches have included antidepressants, antipsychotics, benzodiazepines, nutritional therapy, and immunologic approaches (Santoro et al., 2023).

Recent studies have indicated that a significant number of patients with DSRD also meet diagnostic criteria for catatonia (American Psychiatric Association, 2022; Ghaziuddin et al., 2015; Jakubowicz et al., 2023; Rosso et al., 2020). In many cases, catatonia is a treatable psychomotor and affective condition with distinct physical examination findings, which can be associated with high support needs and affects individuals across the neurodivergent spectrum and lifespan (Hirjak et al. 2024; Luccarelli et al. 2024a, b, c, 2025a, b, c; Luccarelli et al. 2025a, b; Rogers et al. 2023; Smith et al. 2024; Smith et al. 2025a, b; Srinivasan et al. 2024). Broadly, catatonia has been associated with genetic, medical, neurologic, psychiatric, and neurodevelopmental conditions, as well as a high degree of morbidity and mortality especially among children and adolescents (Baldwin et al. 2024; Connell et al. 2023; Hickox et al. 2023; Luccarelli et al. 2022a, b; Raffin et al. 2018; Rogers et al. 2023; Smith et al. 2025a, b). Individuals with neurodevelopmental disorders are at elevated risk for a delayed catatonia diagnosis (Zappia et al., 2024). They may also present with unique catatonic symptoms including loss of previously acquired skills and loss of communicative abilities (Benarous et al. 2018; Smith et al. 2025a, b; Vaquerizo-Serrano et al. 2022), some of which mirror phenotypic aspects of DSRD (Jakubowicz et al., 2023; Smith et al., 2024a).

First-line treatments of catatonia include benzodiazepines and electroconvulsive therapy (ECT) (Hirjak et al. 2024; Luccarelli et al. 2024a, b, c). For neurotypical and neurodivergent individuals across the lifespan, multiple studies of catatonia report high response rates of ECT, even in cases where benzodiazepines have been ineffective (Rogers et al. 2023; Smith et al. 2023, 2024a, b, c, d; Wachtel et al. 2024). In DSRD specifically, smaller case series and reports have shown rapid and effective treatment of catatonia with ECT (Ghaziuddin et al., 2015; Miles et al., 2019). However, these studies are case reports and case series; thus, more data is needed to further solidify the use of ECT for patients with DSRD with co-morbid catatonia symptoms. In this study, we aim to investigate the effectiveness of ECT for DSRD and to report the relative effectiveness of varying doses, frequencies, and types of ECT administered. We report a case series, conduct a systematic review, and combine the data from individual studies with our case series data to conduct an analysis. This approach was chosen given as raw data was obtained from the studies, which differs from meta-analytic approaches of combining study-level summary statistics (Eisenhauer, 2021).

## Methods

### Case Series

The Vanderbilt University Medical Center (VUMC) Institutional Review Board approved this study with a waiver of informed consent from participants (#231964). The clinical records at VUMC were retrospectively queried for patients with a diagnosis of DSRD with catatonic symptoms who received ECT treatments between 7/1/2021 and 5/23/2024. Patients were included in the study if they had clinical diagnoses of DSRD/DSDD and catatonia based on international consensus guidelines and DSM-5 criteria, respectively (American Psychiatric Association, 2022; Santoro et al. 2022a, b). They also required an acute series of ECT treatments (defined as receiving ECT at a frequency of one or more treatments per week), completed a total of more than two ECT treatments as more than one ECT treatment is generally required for clinical response, and had a catatonia assessment measure completed prior to receiving ECT treatments. Subjective medical documentation, physical examination findings, and results of Bush-Francis Catatonia Rating Scale (BFCRS) (Bush et al., 1996) were reviewed for features of DSRD and symptoms of catatonia. The BFCRS is a 23-item measure which grades the severity of each catatonic sign with a maximum score of 3 per item, and a maximum possible BFCRS score of 69 (Bush et al., 1996).

Demographics, past and current medications, psychiatric co-morbidities, scores on the BFCRS, and ECT treatment parameters were extracted from the clinical records. ECT data also included the number of acute treatments (one or more treatments per week) and maintenance treatments (fewer than one treatment per week), EEG seizure duration, and medications given for treatment of catatonia in DSRD.

### Systematic Review

**Fig. 1:**
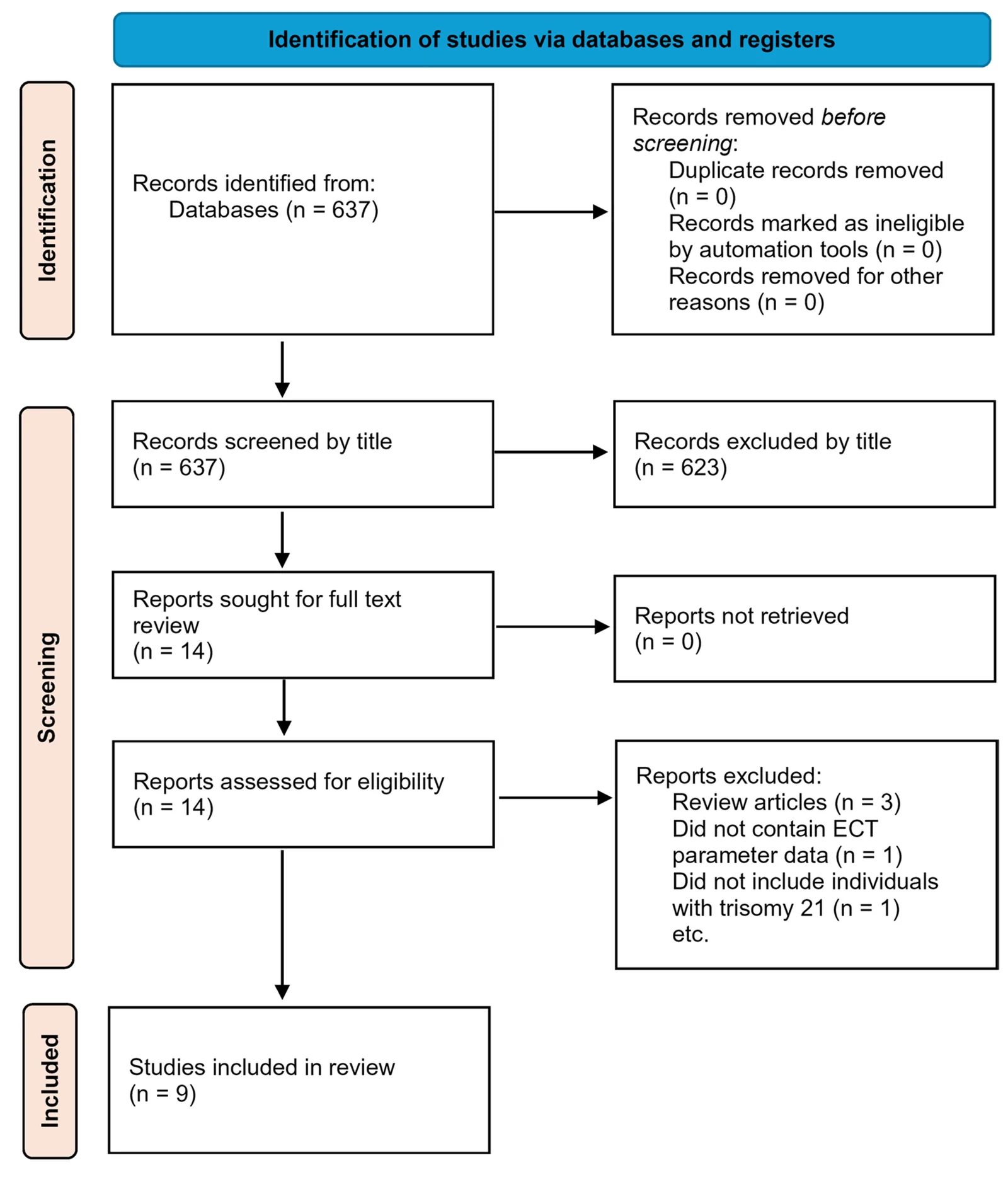
PRIMSA flow diagram

Preferred Reporting Items for Systematic reviews and Meta-Analyses (PRISMA) of peer-reviewed international literature (Fig. 1) was completed to identify additional patients in the literature with DSRD/DSDD and catatonia who received ECT. We identified publications in PubMed with no lower limit on the date of publication until December 16th, 2024. We searched the PubMed database using the following terms: “catatonia,” “down syndrome regressive disorder,” “down syndrome disintegrative disorder,” “trisomy 21,” “electroconvulsive therapy,” and “ECT.” Title review was conducted with ASreview, an open-source machine learning framework for efficient and transparent systematic reviews (Van De Schoot et al., 2021). After title review, abstract review was conducted, then data was extracted manually from identified reports by the research team. Participant-level data was extracted from the identified reports. In instances where participant-level data was unavailable, the study authors were contacted for these data.

### Statistical Analysis

To estimate the main effect of ECT on the overall severity of catatonia in DSRD, we used generalized estimating equations (GEE) (Liang & Zeger, 1986) to estimate the reduction in the mean BFCRS scores before and after ECT while accounting for the correlation between subjects from the same study. BFCRS scores were obtained from the VUMC case series and systematic review. The GEE was specified to have identity linkage function and “independence” working correlation structure and used robust standard error for inference. The results from the GEE were presented as estimated coefficients with 95% confidence interval (CI) and *p*-values. A significance level of 0.05 was used to determine the statistical significance of an estimated effect. The GEE was estimated using the “geepack” package in R (version 1.3.9) (Halekoh et al., 2006) and all statistical analysis of this study were conducted in R version 4.4.1.

## Results

### Systematic Review

As per Fig. 1, our initial search revealed 637 articles, all of which were identified from PubMed. No duplicates were removed. 623 articles were removed by title screening using ASreview software to organize title review via machine learning (Van De Schoot et al., 2021). Reviewers (SL and JRS) manually confirmed exclusion by reviewing all article titles. Fourteen articles underwent full text review. Three were removed as they were review articles, one was removed as it did not contain any ECT data, and one did not contain data for individuals with trisomy 21. A total of 9 studies were identified as meeting criteria for inclusion in the review (Anthonio et al., 2022; Connors et al., 2024; Ghaziuddin et al., 2015; Jap & Ghaziuddin, 2011; Kelley et al., 2024; Miles et al., 2019; Nejati & Etches, 2024; S. L. Santoro et al. 2022a, b; Torr and D’Abrera 2014). The article by Santoro and colleagues did not contain specific ECT data (S. L. Santoro et al. 2022a, b). However, these individuals are co-authors of this manuscript and were able to provide de-identified and updated data for five cases per Massachusetts General IRB #2012P000828. Overall, 22 patients were identified via systematic review. A summary of articles reviewed are contained in supplementary data.

### Study Population

Based on our criteria, 5 patients with DSRD presenting for ECT at VUMC were identified. When combining case series data from VUMC and systematic review, a total of 27 patients were identified and included in the participant-level analysis.

### Demographics

Of patients identified at VUMC, 3 were biologically male and 2 were biologically female. They were all identified as white. The average age was 21.2 years with a minimum of 13 years and a maximum of 32 years. Of the patients identified via systematic review, 10 patients were biologically male and 12 were biologically female. Ten patients were identified as white, 1 was biracial, and 11 were not stated. All patients from the review had age reported with one patient reported as in their “late teens.” The average age of patients with DSRD (*n* = 22) presenting for ECT was 20.6 years with a minimum of 14 years and a maximum of 33 years.

Per Table 1, of all patients with DSRD who received ECT, 14 (51.9%) were female and 13 (48.1%) were male. The mean age was 20.7 years (SD = 5.6). Fifteen (55.6%) patients were white, 1 (3.7%) was biracial, and 11 (40.7%) were unknown.

### Psychiatric and Medical Co-morbidities

Consistent with previous literature, psychiatric and medical comorbidities were common (Table 2). Regarding psychiatric co-morbidities and symptomology, major depressive disorder was present in 4 patients (14.8%). Unspecified mood disorder and autism spectrum disorder affected 3 patients (11.1%), while self-injury was reported in 2 patients (7.4%). Obsessive compulsive disorder, bipolar disorder, unspecified psychosis, post-traumatic stress disorder, tic disorder, and dysthymia were each observed in 1 patient (3.7%).

Hypothyroidism was present in 6 patients (22.2%). Myopia/hyperopia and celiac disease were reported in 4 patients (14.8%). Growth hormone deficiency was found in 3 patients (11.1%), and autoimmune encephalitis and Hashimoto’s thyroiditis were each observed in 2 patients (7.4%). 16p13.11p12.3 duplication was reported in 1 patient (3.7%). Other medical conditions, each affecting 1 patient (3.7%), included keratoconus, cataracts, focal encephalomalacia, aphasia and apraxia, thromboembolism, epilepsy, Hirschsprung’s disease, Crohn’s disease, sick sinus syndrome, cerebral palsy, sleep apnea, and osteopenia.

### Previous Psychopharmacologic Trials for Catatonia in DSRD Before Starting ECT

For all patients included in the analysis, multiple psychopharmacologic trials were attempted for catatonia in DSRD prior to starting ECT, as detailed in Table 3. Among antidepressants, sertraline was administered to 11 patients (40.7%), fluoxetine to 7 patients (25.9%), and citalopram, escitalopram, mirtazapine, trazodone, fluvoxamine, and venlafaxine were each used in 2 patients (7.4%). Bupropion, temazepam, amitriptyline, and duloxetine were each trialed in 1 patient (3.7%).

For antipsychotic medications, quetiapine was given to 3 patients (11.1%) while aripiprazole, ziprasidone, and risperidone were each administered to 2 patients (7.4%). Haloperidol was used in 1 patient (3.7%). Lithium was the mood stabilizer trialed in 2 patients (7.4%). Regarding benzodiazepines, lorazepam was used in 18 patients (66.7%), clonazepam in 2 patients (7.4%), and diazepam and clobazam were each used in 1 patient (3.7%). Among anticonvulsants, valproate and lamotrigine were each administered to 2 patients (7.4%) while ethosuximide was used in 1 patient (3.7%). Zolpidem, a non-benzodiazepine sedative hypnotic, was given to 1 patient (3.7%).

Memantine, an NMDA receptor antagonist, was trialed in 7 patients (25.9%). Alpha agonists clonidine and guanfacine were used in 3 (11.1%) and 2 patients (7.4%), respectively. The number of failed psychiatric medications across the sample was 27, with a mean of 2.4 (SD = 2.6) and a median of 1.

### Active Pharmacologic Treatment for Catatonia in DSRD While Patients Received ECT

Psychopharmacologic regimens for patients with catatonia and DSRD undergoing ECT were recorded for 13 patients, as detailed in Table 4. During ECT treatment, lorazepam was prescribed to 12 patients (44.4%) with varying doses. Fluoxetine was administered to 3 patients (11.1%) while duloxetine and lithium were each prescribed to 2 patients (7.4%). Other active medications during ECT included aripiprazole, lamotrigine, mirtazapine, memantine, escitalopram, famotidine, dextroamphetamine, diazepam, and clobazam, each given to 1 patient (3.7%). From an immunologic perspective, 9 patients (33%) received intravenous immunoglobin (IVIG), and 1 patient (3.7%) received steroids as part of their treatment regimen during ECT.

### ECT Scheduling, Parameters, Anesthetic Dosing, and Adverse Events

Among the 27 patients included in our study, 17 (63.0%) received bitemporal ECT placement while one received bifrontal (3.7%), and the placement for the remaining 9 (33.3%) was unknown. No right unilateral electrode placement was reported. 22 patients had the total number of ECT treatments documented; on average they underwent a total of 52.1 total ECT treatments (SD = 56.1). Ten out of 27 patients reported receiving acute ECT, with an average of 16.8 acute ECT treatments (SD = 76.4). Nine out of 27 patients reported that they received an average of 12.8 maintenance ECT treatments (SD = 16.4). Specific timelines of maintenance treatments were not consistently reported.

ECT data was available for all 5 VUMC patients. All VUMC patients received bitemporal ECT over the course of their treatment. At the time of this report, 4 of the 5 patients had transitioned to maintenance ECT with one remaining in acute treatment. In total, 171 ECT treatments were administered to these 5 VUMC patients over the study period. The mean seizure duration as measured via electroencephalogram for all treatments was 60.9 s (SD = 65.4), with a median of 33 s. In 29 (19%) treatments, propofol was used to terminate prolonged seizure, defined at VUMC as a seizure lasting longer than 2 min. 12 (7.0%) treatments were associated with seizures lasting more than 180 s. The mean energy was 15.02 joules (SD = 8.15). The mean charge was 89.9 mC (SD = 61.7), and the mean frequency was 16 Hz (SD = 5.48). Brief pulse was used in all cases.

Methohexital anesthetic and succinylcholine muscle relaxation were used in all cases. The mean dosage of methohexital was 68.4 mg (SD = 15.5), with a median of 60 mg. The mean dosage of succinylcholine was 51.8 mg (SD = 12.0), with a median of 50 mg. In 27 (15.8%) treatments, intramuscular ketamine was required to obtain intravenous access due to agitation and/or patient distress. The mean dosage of intramuscular ketamine used was 98.9 mg (SD = 20.4), with a median of 100 mg. All 5 patients had difficulties verbalizing subjective side effects as a result of their intellectual disability. However, over the study period, there were no documented significant adverse events related to ECT.

The ECT treatments documented in the systematic review did not have detailed reporting of ECT scheduling, parameters, and anesthetic dosing. Seventeen out of the 22 systematic review patients had the total number of ECT treatments documented. The mean number of total ECT treatments received was 59.2 (SD = 55.6) with a median of 53. Nineteen patients were reported to have transitioned to maintenance ECT treatment.

### Clinical Assessment Measures for Catatonia

Fourteen out of the 22 patients from the systematic review had at least one of pre- and post-treatment BFCRS scores. Ten of the 22 patients had both pre- and post-treatment scores documented; the mean pre-ECT BFCRS score was 18.15 (SD = 6.01), and the mean post-ECT BFCRS score was 4.27 (SD = 6.05).

Combining all patients from VUMC and the systematic review, there were 32 visits with observed BFCRS scores in total (17 pre-ECT and 15 post-ECT) from 18 patients from 7 sites/studies (where VUMC was considered one site). The mean pre-ECT BFCRS score from all patients was 17.35 (SD = 5.72) and the mean post-ECT BFCRS score was 4.40 (SD = 5.26). The GEE analysis revealed that there was a significant difference in the expected BFCRS score before and after receiving ECT treatment (*β* = −13.00 with 95% CI = [−17.70, −8.18], SE = 2.43, *χ* ^2^=28.30, df = 1, *p* < 0.001), suggesting that the ECT treatment had a significant effect on reducing the BFCRS scores.

## Discussion

Our systematic review found that patients with DSRD with co-morbid catatonia responded well to ECT. ECT was shown to reduce catatonic symptoms in 14 patients with DSRD according to the reduction of BFCRS scores. However, it is important to note that there were patients who also received other treatments prior to and during the course of ECT. Therefore, it would be useful to see if other treatment modalities such as IVIG and psychiatric medications improve DSRD and catatonia in future studies.

ECT and benzodiazepines are the gold standard for catatonia treatment across the lifespan for both neurodiverse and neurotypical populations (Rogers et al., 2023; Smith et al., 2022, 2023). In DSRD, clinical response to ECT, benzodiazepines, and other modalities have been reported in case reports and retrospective analyses in small numbers (Ghaziuddin et al., 2015; Jap & Ghaziuddin, 2011; Miles et al., 2019; Santoro et al. 2022a, b; Torr and D’Abrera 2014). To our knowledge, this is the largest analysis done thus far on ECT as a treatment modality for patients with DSRD and catatonia. This is also the first report of ketamine use for IV placement for ECT in DSRD patient with catatonia. The use of ketamine for these purposes has been discussed in the catatonia in autism literature (Srinivasan et al., 2025). Additionally, it is notable that prolonged seizures were relatively common. This is a well-documented side effect of pediatric ECT and thus, likely elevated given the higher number of pediatric patients included in this study (Ghaziuddin et al. 2020; Karl et al. 2022; Puffer et al. 2016; Smith et al. 2024a, b, c, d).

Similar to previous research in the field, we also found a high degree of medical and psychiatric co-morbidity in the DSRD population undergoing ECT. Moreover, many patients had failed multiple pharmacologic treatment options prior to initiating ECT and pursued other therapeutic options (ongoing psychopharmacologic treatment, steroids, and IVIG) while also receiving ECT. This is to be expected as the most common prescribed psychotropic medications in DSRD are selective serotonin reuptake inhibitors (SSRIs), benzodiazepines, and antipsychotics. Traditionally, patients with DSRD without co-morbid catatonia are first treated with SSRIs, benzodiazepines, and antipsychotics (Santoro et al., 2023).

Notably, from a diagnostic perspective some authors have questioned if catatonia is a core component of DSRD or if DSRD is a catatonia spectrum illness (Hauptman et al., 2023; Jakubowicz et al., 2023). There are many overlapping features including staring, grimacing, mutism, and rigidity. Despite the well-documented effectiveness of benzodiazepines in catatonia, Jakubowicz and colleagues found a low frequency of benzodiazepine prescriptions for patients with DSRD who met the criteria for catatonia following a retrospective diagnosis. Regarding ECT, in this reported sample, 8 of the patients in our cohort were under the age of 18 years (Jakubowicz et al., 2023). Thus, despite the effectiveness of ECT observed for catatonia in DSRD and other neurodevelopmental conditions, access to ECT would be limited for them due to restrictive state-dependent legislation due to their age. Moreover, despite its status as a life-saving and life-sustaining intervention, access to ECT is limited to many across the world due to stigma, limited education, and provider availability (Ong et al., 2023; Smith et al., 2022).

Limitations of this study include the small overall sample size, as well as the retrospective nature in which data was collected from our case series and the systematic review. One additional limitation includes an inherent bias toward positive clinical impact from ECT. Specifically, subjects included in our study underwent numerous ECT procedures. Thus, it is unlikely that subjects would have undergone treatment unless there was some noted benefit, possibly overestimating the perceived effectiveness of ECT in this population. Moreover, some studies that were included in our analysis had incomplete data further limiting the generalizability of our findings. One specific example includes the ECT parameters that were only consistently reported in the VUMC patients. Additionally, across all patients, there was inconsistent documentation of the BFCRS. Regardless, our sample size is relatively large compared to the current literature addressing catatonia in DSRD. Moreover, we found statistically significant improvements in the BFCRS and ECT was well tolerated, with no serious adverse events reported.

We recommend that future research investigate the use of ECT for catatonia in DSRD prospectively and report detailed ECT parameters. Recommended data collection should include, but not be limited to, country of ECT administration, study design, number of ECT sessions in the acute phase, frequency of acute ECT sessions, use of maintenance ECT, ECT device, ECT pulse width, mean seizure duration, specific rating scales for psychiatric conditions, and standardized cognitive assessment measures. Future research should also consider comparing ECT to these treatment modalities when catatonia is present and further explore when to initiate ECT for this patient population. Moreover, in light of the significant symptomology associated with catatonia, we also recommend future studies address screening and use of telemedicine in catatonia assessments to improve identification of this disease process (Luccarelli et al. 2022a, b, 2024a, b, c, 2025a, b, c). Overall, given the significant improvement in regression and catatonic symptoms among patients with DSRD, ECT should possibly be considered earlier as a course of treatment.

## Supplementary Material

Supplemental Literature Review

## Figures and Tables

**Table 1:** Demographics of all patients (*N* = 27)

	*N*	%
	27	

Sex		
Female	14	51.9
Male	13	48.1
Race		
White	15	55.6
Unknown	11	40.7
Biracial	1	3.7
Ethnicity		
Non-Hispanic	15	55.6
Unknown	12	44.4
Hispanic	0	0

**Table 2:** Medical and psychiatric co-morbidity of all patients

	*N*	%
	27	

Psychiatric co-morbidity and symptomology		
Major depressive disorder	4	14.8
Autism spectrum disorder	3	11.1
Unspecified mood disorder	3	11.1
Self-injury	2	7.4
Obsessive compulsive disorder	1	3.7
Bipolar disorder	1	3.7
Unspecified psychosis	1	3.7
Post-traumatic stress disorder	1	3.7
Tic disorder	1	3.7
Dysthymia	1	3.7
Medical co-morbidity		
Hypothyroidism	6	22.2
Myopia/hyperopia	4	14.8
Celiac disease	4	14.8
Growth hormone deficiency	3	11.1
Autoimmune encephalitis	2	7.4
Hashimoto’s thyroiditis	2	7.4
16p13.11p12.3 duplication	1	3.7
Keratoconus	1	3.7
Cataracts	1	3.7
Focal encephalomalacia	1	3.7
Aphasia and apraxia	1	3.7
Thromboembolism	1	3.7
Epilepsy	1	3.7
Hirschsprung’s disease	1	3.7
Crohn’s disease	1	3.7
Sick sinus syndrome	1	3.7
Cerebral palsy	1	3.7
Sleep apnea	1	3.7
Osteopenia	1	3.7

**Table 3:** Previous psychopharmacologic trials for catatonia in DSRD before starting ECT

	*N*	%
	27	

Antidepressants		
Sertraline	11	40.7
Fluoxetine	7	25.9
Citalopram	2	7.4
Escitalopram	2	7.4
Mirtazapine	2	7.4
Trazodone	2	7.4
Fluvoxamine	2	7.4
Venlafaxine	2	7.4
Bupropion	1	3.7
Temazepam	1	3.7
Amitriptyline	1	3.7
Duloxetine	1	3.7
Antipsychotics		
Quetiapine	3	11.1
Aripiprazole	2	7.4
Ziprasidone	2	7.4
Risperidone	2	7.4
Haloperidol	1	3.7
Mood Stabilizer		
Lithium	2	7.4
Stimulant		
Lisdexamfetamine	2	7.4
Benzodiazepines		
Lorazepam	18	66.7
Clonazepam	2	7.4
Diazepam	1	3.7
Clobazam	1	3.7
Anticonvulsants		
Valproate	2	7.4
Lamotrigine	2	7.4
Ethosuximide	1	3.7
Non-benzodiazepine Sedative Hypnotic		
Zolpidem	1	3.7
NMDA receptor antagonist		
Memantine	7	25.9
Alpha Agonists		
Clonidine	3	11.1
Guanfacine	2	7.4
Number of failed psychiatric medications	27	
Mean	2.4 (SD=2.6)	
Median	1	

**Table 4:** IVIG, steroids, and psychiatric medications during ECT treatment for all patients

	*N*	%
IVIG		
No	18	67
Yes	9	33
Steroids		
No	26	96.3
Yes	1	3.7
Medications	*n* = 13	
Lorazepam	12	44.4
Fluoxetine	3	11.1
Duloxetine	2	7.4
Lithium	2	7.4
Aripiprazole	1	3.7
Lamotrigine	1	3.7
Mirtazapine	1	3.7
Memantine	1	3.7
Escitalopram	1	3.7
Famotidine	1	3.7
Dextroamphetamine	1	3.7
Diazepam	1	3.7
Clobazam	1	3.7

## Abbreviations

DS: Down syndrome
DSRD: Down syndrome regression disorder
DSDD: Down syndrome disintegrative disorder
ECT: Electroconvulsive therapy
VUMC: Vanderbilt University Medical Center
BFCRS: Bush-Francis Catatonia Rating Scale
GEE: Generalized estimating equations
CI: Confidence interval
IVIG: Intravenous immunoglobulin
SSRIs: Serotonin reuptake inhibitors
